# Supplementary material for: HIVprotI: an integrated web based platform for prediction and design of HIV proteins inhibitors
Source: J Cheminform. 2018 Mar 9;10:12. doi: 10.1186/s13321-018-0266-y (PMC5845081; doi:10.1186/s13321-018-0266-y)
Supplement: Supplementary file 2 — Additional file 2. Source code of HIVProtI web server. [file 13321_2018_266_MOESM2_ESM.zip › HIVprotI_Source-code/hivprotI/weka-3-6-6/doc/weka/core/xml/XMLOptions.html]

XMLOptions


---


|  |  |  |
| --- | --- | --- |
| **Overview** \| **Package** \| **Class** \| **Tree** \| **Deprecated** \| **Index** \| **Help** \| **Weka's home** | |  |
| **PREV CLASS**   **NEXT CLASS** | **FRAMES**    **NO FRAMES**     **All Classes** |  |
| SUMMARY: NESTED \| FIELD \| CONSTR \| METHOD | DETAIL: FIELD \| CONSTR \| METHOD |  |


---


## weka.core.xml Class XMLOptions

```
java.lang.Object
  weka.core.xml.XMLOptions
```

**All Implemented Interfaces:**: RevisionHandler

---

``` public class XMLOptions extends java.lang.Object implements RevisionHandler ```

A class for transforming options listed in XML to a regular WEKA command
line string.

**Version:**
:   $Revision: 1.4 $

**Author:**
:   FracPete (fracpete at waikato dot ac dot nz)

---

| **Field Summary** | |
| --- | --- |
| `static java.lang.String` | `ATT_NAME`             the name attribute. |
| `static java.lang.String` | `ATT_TYPE`             the type attribute. |
| `static java.lang.String` | `ATT_VALUE`             the value attribute. |
| `static java.lang.String` | `DOCTYPE`             the DTD for the XML file. |
| `static java.lang.String` | `ROOT_NODE`             the root node. |
| `static java.lang.String` | `TAG_OPTION`             tag for a single option. |
| `static java.lang.String` | `TAG_OPTIONS`             tag for a list of options. |
| `static java.lang.String` | `VAL_TYPE_CLASSIFIER`             a value of the type attribute. |
| `static java.lang.String` | `VAL_TYPE_FLAG`             a value of the type attribute. |
| `static java.lang.String` | `VAL_TYPE_HYPHENS`             a value of the type attribute. |
| `static java.lang.String` | `VAL_TYPE_OPTIONHANDLER`             a value of the type attribute. |
| `static java.lang.String` | `VAL_TYPE_QUOTES`             a value of the type attribute. |
| `static java.lang.String` | `VAL_TYPE_SINGLE`             a value of the type attribute. |


**Constructor Summary**

`XMLOptions()`
  
          Creates a new instance of XMLOptions.

`XMLOptions(java.io.File file)`
  
          Creates a new instance of XMLOptions.

`XMLOptions(java.io.InputStream stream)`
  
          Creates a new instance of XMLOptions.

`XMLOptions(java.io.Reader reader)`
  
          Creates a new instance of XMLOptions.

`XMLOptions(java.lang.String xml)`
  
          Creates a new instance of XMLOptions.


| **Method Summary** | |
| --- | --- |
| `org.w3c.dom.Document` | `getDocument()`             returns the parsed DOM document. |
| `java.lang.String` | `getRevision()`             Returns the revision string. |
| `boolean` | `getValidating()`             returns whether a validating parser is used. |
| `XMLDocument` | `getXMLDocument()`             returns the handler of the XML document. |
| `static void` | `main(java.lang.String[] args)`             for testing only. |
| `void` | `setValidating(boolean validating)`             sets whether to use a validating parser or not. |
| `java.lang.String[]` | `toArray()`             returns the current DOM document as string array. |
| `java.lang.String` | `toCommandLine()`             returns the given DOM document as command line. |
| `java.lang.String` | `toString()`             returns the object in a string representation (as indented XML output). |

**Methods inherited from class java.lang.Object**

`equals, getClass, hashCode, notify, notifyAll, wait, wait, wait`

**Field Detail**

### TAG\_OPTION

```
public static final java.lang.String TAG_OPTION
```

:   tag for a single option.

    **See Also:**: Constant Field Values

---


### TAG\_OPTIONS

```
public static final java.lang.String TAG_OPTIONS
```

:   tag for a list of options.

    **See Also:**: Constant Field Values

---


### ATT\_NAME

```
public static final java.lang.String ATT_NAME
```

:   the name attribute.

    **See Also:**: Constant Field Values

---


### ATT\_TYPE

```
public static final java.lang.String ATT_TYPE
```

:   the type attribute.

    **See Also:**: Constant Field Values

---


### ATT\_VALUE

```
public static final java.lang.String ATT_VALUE
```

:   the value attribute.

    **See Also:**: Constant Field Values

---


### VAL\_TYPE\_FLAG

```
public static final java.lang.String VAL_TYPE_FLAG
```

:   a value of the type attribute.

    **See Also:**: Constant Field Values

---


### VAL\_TYPE\_SINGLE

```
public static final java.lang.String VAL_TYPE_SINGLE
```

:   a value of the type attribute.

    **See Also:**: Constant Field Values

---


### VAL\_TYPE\_HYPHENS

```
public static final java.lang.String VAL_TYPE_HYPHENS
```

:   a value of the type attribute.

    **See Also:**: Constant Field Values

---


### VAL\_TYPE\_QUOTES

```
public static final java.lang.String VAL_TYPE_QUOTES
```

:   a value of the type attribute.

    **See Also:**: Constant Field Values

---


### VAL\_TYPE\_CLASSIFIER

```
public static final java.lang.String VAL_TYPE_CLASSIFIER
```

:   a value of the type attribute.

    **See Also:**: Constant Field Values

---


### VAL\_TYPE\_OPTIONHANDLER

```
public static final java.lang.String VAL_TYPE_OPTIONHANDLER
```

:   a value of the type attribute.

    **See Also:**: Constant Field Values

---


### ROOT\_NODE

```
public static final java.lang.String ROOT_NODE
```

:   the root node.

    **See Also:**: Constant Field Values

---


### DOCTYPE

```
public static final java.lang.String DOCTYPE
```

:   the DTD for the XML file.

    **See Also:**: Constant Field Values


**Constructor Detail**

### XMLOptions

```
public XMLOptions()
           throws java.lang.Exception
```

:   Creates a new instance of XMLOptions.

    **Throws:**: `java.lang.Exception` - if the construction of the DocumentBuilder fails **See Also:**: `setValidating(boolean)`

---


### XMLOptions

```
public XMLOptions(java.lang.String xml)
           throws java.lang.Exception
```

:   Creates a new instance of XMLOptions.

    **Parameters:**: `xml` - the xml to parse (if "xml" is not found then it is considered a file) <DT**Throws:**: `java.lang.Exception` - if the construction of the DocumentBuilder fails **See Also:**: `setValidating(boolean)`

---


### XMLOptions

```
public XMLOptions(java.io.File file)
           throws java.lang.Exception
```

:   Creates a new instance of XMLOptions.

    **Parameters:**: `file` - the XML file to parse **Throws:**: `java.lang.Exception` - if the construction of the DocumentBuilder fails **See Also:**: `setValidating(boolean)`

---


### XMLOptions

```
public XMLOptions(java.io.InputStream stream)
           throws java.lang.Exception
```

:   Creates a new instance of XMLOptions.

    **Parameters:**: `stream` - the XML stream to parse **Throws:**: `java.lang.Exception` - if the construction of the DocumentBuilder fails **See Also:**: `setValidating(boolean)`

---


### XMLOptions

```
public XMLOptions(java.io.Reader reader)
           throws java.lang.Exception
```

:   Creates a new instance of XMLOptions.

    **Parameters:**: `reader` - the XML reader to parse **Throws:**: `java.lang.Exception` - if the construction of the DocumentBuilder fails **See Also:**: `setValidating(boolean)`


**Method Detail**

### getValidating

```
public boolean getValidating()
```

:   returns whether a validating parser is used.

    :   **Returns:**: whether a validating parser is used

---


### setValidating

```
public void setValidating(boolean validating)
                   throws java.lang.Exception
```

:   sets whether to use a validating parser or not.   
    Note: this does clear the current DOM document!

    :   **Parameters:**: `validating` - whether to use a validating parser **Throws:**: `java.lang.Exception` - if the instantiating of the DocumentBuilder fails

---


### getDocument

```
public org.w3c.dom.Document getDocument()
```

:   returns the parsed DOM document.

    :   **Returns:**: the parsed DOM document

---


### getXMLDocument

```
public XMLDocument getXMLDocument()
```

:   returns the handler of the XML document. the internal DOM document can
    be accessed via the `getDocument()` method.

    :   **Returns:**: the object handling the XML document **See Also:**: `getDocument()`

---


### toCommandLine

```
public java.lang.String toCommandLine()
                               throws java.lang.Exception
```

:   returns the given DOM document as command line.

    :   **Returns:**: the document as command line **Throws:**: `java.lang.Exception` - if anything goes wrong initializing the parsing

---


### toArray

```
public java.lang.String[] toArray()
                           throws java.lang.Exception
```

:   returns the current DOM document as string array.

    :   **Returns:**: the document as string array **Throws:**: `java.lang.Exception` - if anything goes wrong initializing the parsing

---


### toString

```
public java.lang.String toString()
```

:   returns the object in a string representation (as indented XML output).

    :   **Overrides:**: `toString` in class `java.lang.Object`
    :   **Returns:**: the object in a string representation

---


### getRevision

```
public java.lang.String getRevision()
```

:   Returns the revision string.

    :   **Specified by:**: `getRevision` in interface `RevisionHandler`
    :   **Returns:**: the revision

---


### main

```
public static void main(java.lang.String[] args)
                 throws java.lang.Exception
```

:   for testing only. prints the given XML file, the resulting commandline and
    the string array.

    :   **Parameters:**: `args` - the commandline options. **Throws:**: `java.lang.Exception` - if something goes wrong


---


|  |  |  |
| --- | --- | --- |
| **Overview** \| **Package** \| **Class** \| **Tree** \| **Deprecated** \| **Index** \| **Help** \| **Weka's home** | |  |
| **PREV CLASS**   **NEXT CLASS** | **FRAMES**    **NO FRAMES**     **All Classes** |  |
| SUMMARY: NESTED \| FIELD \| CONSTR \| METHOD | DETAIL: FIELD \| CONSTR \| METHOD |  |


---
